# Expertise and Deceptive Movements in Sport

**DOI:** 10.1186/s40798-024-00730-8

**Published:** 2024-06-11

**Authors:** Ryan Raffan, David Mann, Geert Savelsbergh

**Affiliations:** 1 Department of Human Movement Science, Nelson Mandela University, Port Elizabeth, South Africa; 2 Department of Human Movement Sciences, Vrije Universiteit Amsterdam, Amsterdam Movement Sciences and Institute Brain and Behavior Amsterdam (iBBA), Amsterdam, The Netherlands; 3 Faculty of Sports and Nutrition, Amsterdam University of Applied Sciences, Amsterdam, The Netherlands

**Keywords:** Deception, Perception, Anticipation, Expertise, Action

## Abstract

**Background:**

Deceptive movements occur when an actor seeks to fake, hide or delay kinematic information about their true movement outcomes. The purpose of deceptive movements is to impair the perception of opponents (the ‘observer’) to gain an advantage over them. We argue though that a lack of conceptual clarity has led to confusion about what deception is and in understanding the different approaches by which an actor can deceive their opponent. The aim of this article is to outline a conceptual framework for understanding deceptive movements in sport.

**Main body:**

Adopting Interpersonal Deception Theory from the field of communication, we define deception as when an actor deliberately alters their actions in an attempt to impair the ability of an observer to anticipate their true action outcomes. Further, deception can be achieved either by what we define as deceit, the act of providing false information, or disguise, the act of concealing the action outcome. Skilled athletes often have actions that are difficult to anticipate, but an action is only classified as containing deception if the actor has explicit intent to deceive an observer. Having outlined the conceptual framework, we then review existing empirical findings on the skilled perception of deceptive movements considering the framework. This approach includes a critical evaluation of the mechanisms known to facilitate the perceptual ability to prevent being deceived, including a consideration of visual search strategies, confidence, the contribution of visual and motor experiences, and the influence of response biases and action capabilities on perceptual performance.

**Conclusion:**

The distinction between deceit and disguise particularly helps to show that most research has examined deceit, with little known about how an actor can more effectively disguise their action, or about how an observer can improve their ability to anticipate the outcome of disguised actions. The insights help to identify fruitful areas for future research and outline implications for skill acquisition and performance enhancement.

## Key Points

- We offer a new conceptual framework for deceptive actions in sport, in which deceitful and disguised actions are a subset of deception, in order to clarify confusion in the existing literature.
- We discuss the role of intent in performing deceptive actions and the implications this might have in seeking to train deception to impair the perceptual ability of an opponent.
- We make predictions about the response accuracies of observers attempting to anticipate the deceitful and disguised action intentions of skilled and less-skilled actors.

## Background

During time-constrained interactive motor tasks such as one-on-one duels in sports including basketball and football, defenders attempt to anticipate the movement intentions of their opponents to defend their space. Equally, the attacker may utilise deceptive movements by faking or hiding their movement intention, aiming to ‘fool’ the defender to negate their anticipatory ability. This interplay between anticipation and deception can continue until one outperforms the other [1, 2]. Understanding this interaction between anticipation and deception has both theoretical and practical relevance [3]. From a broader theoretical standpoint, an understanding of the mechanisms underlying deception is important for helping to comprehend the fundamental mechanisms that underpin action understanding in humans [2, 4, 5]. And from a practical perspective, an understanding of how best to create a deceptive movement – and how to avoid one – is relevant for improving the performance of both actors *and* observers (see [6, 7] for relevant examples).

Despite the omnipresent role that deception plays in many sports, it took a surprisingly long time for research in sport science to address this important issue. Research on the perception of deceptive actions was initially studied *outside* of sports, for instance by Runeson and Frykholm [8], who investigated the ability of observers to detect deceptive actions performed by actors when picking up a box. The authors video recorded inexperienced actors lifting three differently weighted boxes (6.5 kg, 11 kg & 19 kg) from the ground onto a table, with the actors unaware of the weight of the box before lifting it (producing *true or veridical* actions). The actors were then recorded lifting an empty box (4 kg), but while trying to give the impression that the weight of the box was the same as one of the three initial weights (producing a *fake* action). Test videos comprising of both veridical and fake actions were re-created using point-light displays, which removes all the visual features in the environment and displays only points-of-light at key joint centres to purely convey the kinematic information inherent in the action [9]. Naïve observers viewed each video clip and made a judgment about whether they had seen a true or fake movement. Results revealed that observers were very good at detecting the ‘fake’ clips, with it being very difficult for actors to deceive the observers, particularly when excessive deception was involved. What was most remarkable was that observers could clearly distinguish between veridical and deceptive intentions even though they did not necessarily have expertise in observing these types of actions, suggesting that humans have the capability to detect deception even in the absence of experience in performing or observing that task. The results were proposed to have supported the principle of the *Kinematic Specification of Dynamics*, which suggests that kinematic movements specify the natural causal effects of events in a direct manner without the observer necessarily needing to have had experience or expertise in performing or viewing the specific task which they are viewing [8].

While the task of lifting a box might not require specialised skills for perceiving intent, sports provide a situation where skilled performers are known to develop a specialised ability to pick-up kinematic information from the actions of opponents. It could also be that their sensitivity to pick up kinematic information would make them more likely to be susceptible to deception. However, it took more than 20 years after the work of Runeson and Frykholm [8] before the seminal work of Jackson et al. [3] sparked a new field of research in deception and anticipation in sport in an attempt to answer this question. In their study, Jackson et al. [3] examined the susceptibility of skilled and less-skilled rugby players to deceptive movements. Rugby players viewed video footage of opponents who ran towards them and in some cases performed a deceptive side-step, and in other cases did not. Video clips were occluded at predetermined moments in the action sequence, with observers required to anticipate the impending running direction of the opponent following occlusion. Results revealed that skilled observers performed equally well on deceptive and non-deceptive trials while less-skilled observers performed significantly worse than skilled observers on deceptive trials, but not on the non-deceptive trials. These results suggest that experts are *better* able to perceive deceptive intent, and therefore that expertise may rely at least in part on the ability to detect complex deceptive actions in others. From these results, a new field of research has emerged examining both the ability to *perform* deceptive actions in sport in conjunction with the ability to *avoid* those deceptive actions. Given that it has been just over 15 years since the publication of Jackson et al.’s paper, it seems timely to revisit the impact of the paper and to propose extensions that help us to understand what is now known about the role of deception in sport (also see [5, 10]).

## Deceptive Movements (Re-)Defined

**Fig. 1:**
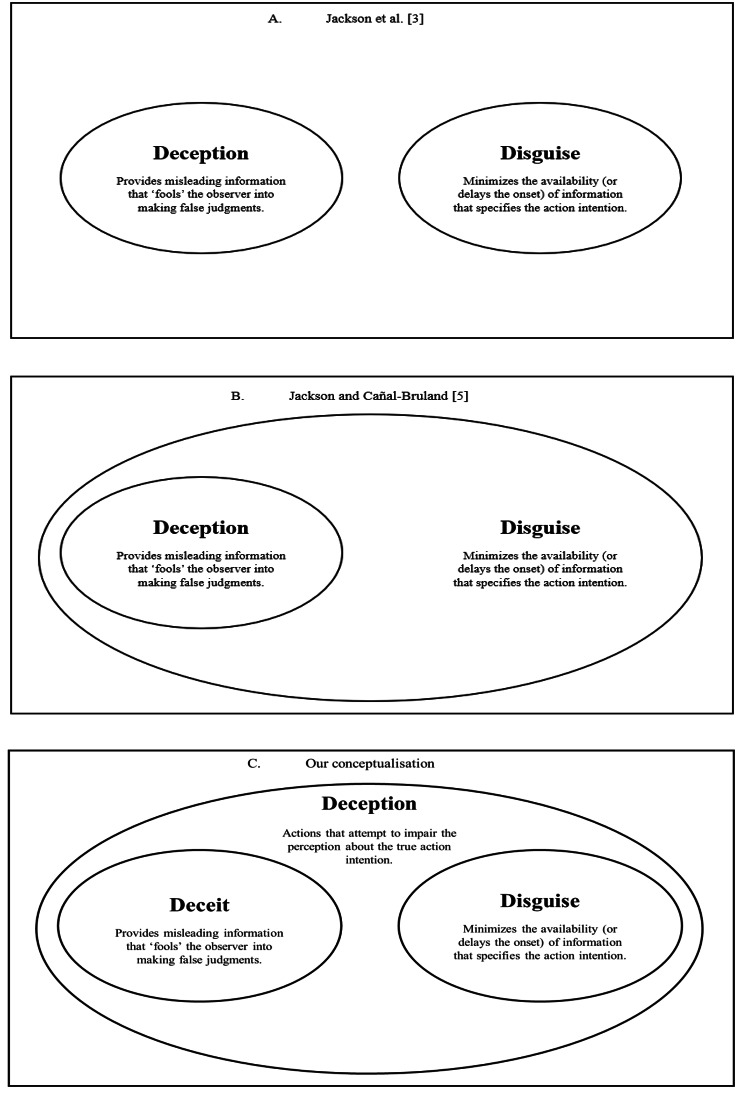
Schematic demonstration of the conceptual framework of deceptive movement in sport. **A**. is the original working definition from Jackson et al. [3]. **B**. is an updated version presented by Jackson and Cañal-Bruland [5]. **C**. is our conceptualisation of deceptive movement in sport with deceit and disguise forming subsets of deception

A clear conceptual framework is necessary for what constitutes a deceptive action, particularly given the confusion that we have observed about key concepts and terminology in the literature. Jackson et al. [3] provided a working definition of deception, making a clear and unambiguous distinction between deception and disguise (see Fig. 1A). *Disguise* was defined as an actor’s attempt to *hide* or *delay* the onset of informative cues to prolong the response of the observer [3]. In contrast, *deception* referred to the provision of *false* information that misled or ‘fooled’ an observer so that they made incorrect judgements [3]. Although this distinction provided a relatively clear differentiation between the two *types* of actions utilized by actors, there has since been confusion about the *terms* used within the field. In particular, there have been conflicting accounts of whether disguise is itself a form of deception. Indeed, some authors have unintentionally conflated the two terms by describing a disguised action as being a form of ‘deception’, or as having a ‘deceptive effect’ [11–15]. Essentially, some in the field appear to be using the term ‘deception’ to refer to any attempt by an actor to impair the ability of an opponent to anticipate their action outcomes, irrespective of whether that is achieved by hiding the true action intent or by providing false information about that action intent. This leads to problems in effectively comparing the approaches and results of different studies in the field of deception.

In 2019, Jackson and Cañal-Bruland [5] presented a new conceptualisation of deception and disguise that sought to clarify the relationship between the two. Instead of treating deception and disguise as separate entities, or disguise as a sub-set of deception, they conceptualised deception as a sub-set of disguise (see Fig. 1B). This approach was advantageous in that it acknowledged that both the act to *hide* intent and the act to *convey false* intent were both manners by which an actor could circumvent an opponent’s ability to anticipate the actor’s true action intentions. However, this modification was in contrast to how some in the literature appeared to be treating disguise as a form of deception [11–15]. Moreover, it introduced further confusion over the term ‘disguise’. By repositioning the term ‘disguise’ to refer to both the act to hide *and* to convey false intent, it means that there was now no exclusive label for the act to minimize the availability of kinematic information (what Jackson et al. [3]. referred to as ‘disguise’) in the absence of false information that ‘fools’ the opponent into making a false judgement (what Jackson et al. [3]. referred to as ‘deception’). Moreover, within their conceptualisation, ‘disguise’ was still defined as an act that reduces an opponent’s response accuracy to ‘chance levels’ rather than producing incorrect responses (see Jackson & Cañal-Bruland [5], Fig. 6.1). When someone refers to ‘disguise’ within this framework, what are they referring to? Hiding intent? False intent? Or both? If we accept that ‘misleading kinematic information’ and ‘delayed kinematic information’ are separate entities (as Jackson et al. [3]. , did for deception and disguise; see Fig. 1A), and that both are subsets of some larger entity describing ‘an attempt to impair perception’ (as Jackson & Cañal-Bruland [5] appear to do; see Fig. 1B), then we argue that concepts to ‘mislead’, ‘delay’, and ‘impair’ should all have their own unambiguous labels (see Fig. 1C).

To address this confusion and to help establish a clearer conceptual framework of deceptive movements in sport, we draw on the *Interpersonal Deception Theory* of deceptive communication proposed by Buller and Burgoon [16] to help with some of the terminology and definitions used within sport. Interpersonal Deception Theory is based on the premise that people in conversation with each other expect the other person to conform to pro-social behaviour by telling the truth. If one of the interacting persons violates this expectancy with the use of deception, then the other person may become suspicious and therefore becomes unsure whether information is truthful or deceptive. Moreover, Interpersonal Deception Theory defines deception as a sender’s *deliberate* act that is intended to alter the interpretation of the receiver. This definition highlights two cornerstones of deception: that deception alters perception, and that it is intentional. Consequently, deception is considered an umbrella term which encompasses three strategic forms: an attempt to *hide* the truth (*concealment*); an attempt to *lie* about the truth (*falsification*); and an attempt to *avoid* a point altogether (*equivocation*).

To what degree does Interpersonal Deception Theory apply to actions performed in sports? As we are about to argue, some aspects such as the terminology and definitions are very useful and we believe help to resolve some of the confusion in the literature in the sport sciences. On the other hand, there are limitations. For instance, the expectation in Interpersonal Deception Theory that people in conversation tell the truth does not necessarily hold in sport: athletes would typically expect opponents to actively deceive them. This might mean that athletes are inherently more sceptical in their interactions in sport and that there is more motivation for athletes to be able to detect and see through deceptive intent. Moreover, there are aspects of inter-personal communication that do not necessarily occur in movements. We revisit this shortly too. We do not seek to translate Interpersonal Deception Theory into sports in its entirety, but nonetheless there are several clear benefits and lessons that can improve the theoretical understanding of deception in sports.

First, Interpersonal Deception Theory can be applied to help clarify the terms used for deceptive actions within sports. Deception, as applied to movements in sports, would refer to when an actor deliberately alters their behaviour in an attempt to impair the ability of an observer to anticipate their true action outcome. Deception could be achieved by the actor attempting to either conceal *or* falsify their true/veridical movements. The act of hiding or delaying veridical movement information would be *concealment*, and the act of providing false movement information about the true action outcome would be classed as *falsification*. Equivocation, which is when a communicator avoids a point altogether, is unlikely to apply to competitive sport given that it is difficult to simply avoid a point or interaction altogether. Finally, a veridical (non-deceptive) action is one where the actor performs their typical action without any attempt to hide/delay or provide false information about their action outcome. According to Interpersonal Deception Theory, a veridical action would be the equivalent of *truth telling*.

Accordingly, our conceptualisation of deceptive actions contains three key concepts (see Fig. 1C). First, by adopting Interpersonal Deception Theory, we continue to label *disguise* as the act of hiding or concealing an action intention in the absence of any falsification of the true action intention. Second, we define *deceit* as the act of providing false intention about the true action outcome. Third, we consider both concepts to be subsets of *deception*, that is, any act that seeks to deliberately impair the ability of the opponent to anticipate an actor’s true action outcome. This conceptualisation is advantageous in that it applies an established scientific theory of deception to the field of deception in sport, and in that it provides three unambiguous terms for the concepts being addressed in the scientific literature on deception in sport. Moreover, the conceptualisation uses the term ‘deception’ in a manner that appears to be more consistent with how it is being used within the scientific literature in sport (e.g [11–15, 17, 18]). This overarching definition of deception would encompass all synonyms of deception such as when performing a ‘fake’, ‘feint’, ‘trick’, or ‘bluff’.

How are these deceptive movements typically produced? Deceit is typically generated by exaggerating critical kinematic cues, by altering the angle of approach, or by presenting misleading postural gestures (such as the head or gaze direction) that convey an outcome that is different to that which ultimately occurs [3]. For example, a rugby player may exaggerate their evasive side-stepping manoeuvres to give the impression that they will move in the opposite direction to that which they ultimately take (see [12]). An example of a misleading postural gesture is when a penalty taker in football or handball uses a posture with the head and/or gaze direction facing one way, but with the intention of kicking or throwing the ball in the opposite direction (see [13, 15, 19, 20] for examples). Further, a footballer may seek to fool a referee into calling a foul or penalty by exaggerating the effect of an opponent’s actions on their own movement [21–23]. This latter case is an important one because it is in many cases an example of an actor exaggerating the effect of a veridical action (e.g., making a small trip look much more significant than it really is) rather than conveying an action outcome that is the opposite to that which will ultimately occur (e.g., in a rugby side-step). These exaggerated actions still fit within the definition of deception as outlined by Interpersonal Deception Theory.

Disguise, on the other hand, is created by minimizing or delaying the availability of veridical kinematic information that specifies the intended action outcome [3]. For example, a volleyball player may hide/delay the intention to perform a smash by giving the impression that either a smash or poke-shot could be performed [24]. Effective disguise may require an actor to develop action capabilities that allow them to change their direction of movement as quickly and as late as possible [25].

A crucial question when seeking to apply Interpersonal Deception Theory to sports is whether an actor needs to have an explicit *intent* to be deceptive for an action to be considered ‘deceptive’. The most significant challenge to this question is perhaps in deciding what constitutes a ‘disguised’ action. For instance, many skilled athletes have actions that are ‘difficult-to-anticipate’, often because they have action capabilities that allow them to produce rapid changes in movement direction as late as possible. Those athletes might be commonly described as having ‘well-disguised’ actions from the perspective of the observer viewing the action, but can those actions be classified as ‘disguised’ from a theoretical standpoint? According to Interpersonal Deception Theory, the difficult-to-anticipate actions should not be classified as disguised if they do not differ from the actor’s ‘regular’ veridical movements and were not performed with an intent to impair the perception of the opponent. Instead, we simply need to accept that some actors have actions that are more difficult to anticipate than others [26] and that they should not be classified as ‘disguised’ actions. If we were to classify some veridical but difficult-to-anticipate actions as ‘disguised’, then what criteria would we use to decide what made an action ‘disguised’ as opposed to ‘veridical’? This could be done on the basis of the ability of opponents to anticipate those actions. But this is problematic. First, some opponents are clearly better at anticipating than others [27], so which observers would we use as our reference point? Second, what would be our cut-off point when deciding that an action was ‘disguised’? Instead, it appears more logical to distinguish veridical/non-deceptive actions from disguised actions on the basis of the intent on the part of the actor. If the actor modifies their action with the intent to hide their action intention then the action should, consistent with Interpersonal Deception Theory, be considered as disguised. The inclusion of intent appears to be the best way to distinguish disguised from veridical actions.

A deliberate effort to train an action to become more ‘disguised’ presents an interesting challenge to the requirement for ‘intent’ in the definition of deception [28]. For example, Pete Sampras’s tennis serve was notoriously difficult to anticipate, reportedly because his junior coach during service drills would call out the required direction of serve only late in the service action [29]. This seemingly resulted in specifying information about the actual direction of the serve being available only very late in the service action, effectively resulting in a highly ‘disguised’ action (see [8]). However, it is doubtful that this form of ‘disguise’ was performed *deliberately* each time Sampras served later in life. Instead, it was probably implicit within Sampras’s action and he had very little deliberate intent to hide any specifying kinematic information. Should Sampras’s serve be classified as ‘disguised’? The answer from the viewpoint of Interpersonal Deception Theory would be ‘no’, because there was no deliberate intent to impair the perception of the opponent at that moment. Conceptually this is challenging: there was intent on the part of the coach to produce an action that was well disguised, but presumably Sampras had no intent to disguise each time he served.

Turning to deceit, what if an action conveys deceit but there is no intent on the part of the actor to convey that deceit? Is that even likely? It is possible to conceive of situations, for instance when an actor produces an action that was intended to be veridical but was erroneously deceitful. For example, an actor such as a football penalty-taker could lose balance when performing an action, and that change in action could be perceived by the goalkeeper to be a kick going in the opposite direction to what the kicker intended (e.g., going left when it was intended to go right). In that case, according to Interpersonal Deception Theory, the action would not be classed as being deceit because the kicker did not intent to be deceitful, even if it did deceive the goalkeeper. Instead, Interpersonal Deception Theory would call such an act an *honest mistake*. In such instances, the performer did not act deliberately, but perception may have been impaired.

So when deciding whether an action is deceptive or not, it is important to decide from the standpoint of the *actor* performing the action, and not from the standpoint of the observer viewing that action. As we have seen, an action can be deceptive from the perspective of the observer, but not the actor (e.g., when an actor performs an erroneous action). Similarly, a skilled athlete with a difficult-to-anticipate action might to the observer appear well ‘disguised’ even though the actor had no intent to deliberately disguise their action at that moment. In these cases, the actions would not, according to Interpersonal Deception Theory, be classified as deception, because they lacked intent on the part of the actor to deceive, irrespective of whether the observer was deceived. Conversely, an action can be deceptive from the perspective of the actor, but not the observer, for example when an actor performs an action that they intend to be disguised or deceitful, but it was in no way effective in impairing the ability of the observer to predict the action intention (e.g., when the actor is first learning to deceive). Or when an action is deceptive but the observer lacks the skill or experience to associate the deceptive intent with the likely action outcome. Those actions would, according to Interpersonal Deception Theory, be classified as deception even though they did not deceive the observer. Clearly there is not a one-to-one relationship between what the actor intends and what the observer perceives, and this will no doubt continue to cause some confusion, and perhaps even doubt about whether the actor’s intent should remain a vital element of deciding whether an action is deceptive or not.

## Susceptibility of Skilled Observers to those Actions

The seminal work of Jackson et al. [3] investigated the anticipation of skilled observers and their susceptibility to what we would now more specifically classify as *deceitful* actions. Their finding of similar anticipatory accuracy for skilled and less-skilled observers when viewing veridical (non-deceptive) movements is surprising given the usual finding of an expert advantage for anticipation. The authors suggested that the perception of non-deceptive running actions might have been a generalisable enough skill in which the less-skilled observers had sufficient perceptual experience from viewing biological motion in everyday activities [8]. In the presence of deceitful actions, though, skilled observers significantly outperformed their less-skilled counterparts. Only the performance of the less-skilled observers dropped when viewing deceitful movements; skilled observers performed equally well on both deceitful and non-deceptive trials. The authors concluded that only less-skilled observers were susceptible to deceitful actions. Mori and Shimada [30] replicated the study of Jackson et al. [3], finding that skilled observers do significantly outperform less-skilled observers when viewing deceitful movements, but that the skilled observers *were* susceptible to deceitful actions, though to a lesser degree than lesser-skilled observers [22, 31–34]. More recently, it has been demonstrated that skilled observers are more capable of quickly learning to overcome a susceptibility to deceptive intent than lesser-skilled observers are [35].

Deceitful bodily actions are effective for deceiving an opponent, but as noted, there are other cues such as head fakes and the direction of gaze which could convey deceptive intent. Mann et al. [20] investigated whether an actor’s gaze direction itself could be used as an effective cue for deception. Skilled and less-skilled football players attempted to intercept a pass kicked in situ to either their right or left. The gaze direction of the kicker provided no clue to the movement outcome: in half of the trials the kicker’s gaze aligned with the kick direction, whereas in the remaining trials gaze was directed towards the opposite direction. The results showed that the performance of both the skilled and lesser-skilled groups dropped in the presence of deceitful gaze, but that the performance of the less-skilled observers dropped significantly more than the skilled observers. The authors suggested that the head and/or gaze direction can be a powerful non-kinematic cue for deception (also see [15, 19] for relevant examples). This highlights the point that more than simply kinematic information is used to convey deceptive intent in sport situations, and that these ‘head feints’ and ‘no look passes’ should be classified as deceptive if they are performed with deliberate intent on the part of the actor.

Rowe et al. [11] examined the influence of *disguised* actions on the perception of skilled observers. Skilled and less-skilled tennis players watched temporally occluded video footage of actors producing disguised and veridical (non-deceptive) forehand and backhand ground strokes. Results revealed a significant advantage for the skilled observers when viewing non-deceptive strokes, but when disguise was present, this anticipatory advantage for skilled observers in some situations disappeared. If disguise occurred early in the action sequence, then the skilled observers maintained their anticipatory advantage by the moment of racquet-ball contact. However, if disguise could be maintained later in the action sequence then the anticipatory advantage of the skilled observers was reduced, indicating that skilled observers are susceptible to actions that are disguised late in the action sequence, suggesting that a skilled actor’s superior action capabilities can indeed be advantageous for deceiving skilled opponents. The authors concluded that disguised actions can be an effective deceptive strategy for delaying the perception of observers and disrupting anticipatory performance (also see [24]).

An alternative approach to disguising actions themselves is to manipulate the *uniform* worn by actors in an attempt to conceal the veridical information that may specify their action outcome. For example, Causer and Williams [14] filmed actors performing non-deceptive football penalty kicks with the kickers wearing either neutral or patterned (circled or zigzagged) shirts. Skilled and less-skilled observers were required to anticipate the intended direction of the penalty kick while watching a temporally occluded video. Again, skilled observers outperformed their less-skilled counterparts. However, the anticipatory advantage of the skilled observers diminished when anticipating the patterned shirts, particularly later in the action sequence. The authors concluded that the uniform design could have potential benefits for successfully disguising the kinematics of actors and reducing the anticipatory accuracy of skilled opponents.

Research into deception in sports remains in its infancy and there is much still to learn. Studies have begun to investigate the impact of deception in in-situ scenarios [20] and during competition [36, 37], but much more is needed to understand whether the effects found in more controlled laboratory-based studies are representative of those found during competition. Moreover, the majority of existing studies have been designed using one-on-one situations with ‘passive’ observers, which contrasts with the real-life situations experts find themselves in (e.g., see [36, 37]). Future research should consider unlocking the ‘passive’ observer to allow interaction between the actor and observer [38﻿] as well as extending the deceptive situation to include multiple interacting players. Additionally, training actors to disguise their actions may be a worthwhile endeavour given the adverse effect on the perception of skilled observers late in the action sequence.

## Mechanisms Underpinning the Perception of Deceptive Actions

Understanding the mechanisms that support the ability to ‘see through’ deceptive intent could allow researchers to manipulate training to accelerate skill acquisition and enhance anticipatory performance. In this section, underlying concepts such as visual search strategies, confidence ratings, and visual and motor experience are discussed along with some interpretation and reflections based on the application of Interpersonal Deception Theory.

### Visual Search Strategies to Anticipate Deceptive Actions

In an extension of the study by Jackson et al. [3], Mori and Shimada [30] fitted an eye-tracker to skilled and less-skilled rugby players who watched video footage of skilled opponents performing deceitful side-stepping manoeuvres. Irrespective of the action being viewed, skilled observers spent significantly more time focusing on body segments such as the hips and legs that presumably specify the action outcome (also see [34] for similar body segments in basketball). In contrast, less-skilled observers generally focused more on the opponent’s chest, particularly when opponents performed a deceitful action. The authors concluded that skilled observers attuned to veridical information whereas the attention of the less-skilled observers was drawn to the distracting deceitful information conveyed by the actors (also see [31]). Interestingly, recent work by Meyer et al. [39] found that both skilled and less-skilled defensive players in basketball were vulnerable to deceitful actions by focusing on specific areas (such as the head or ball) even after being instructed by expert coaches to focus on the torso and on the importance of using peripheral vision. These findings highlight the deliberate intent of actors to utilise exaggerations within their deceitful actions to attract the attention of observers away from veridical kinematic information. In addition, the effectiveness of explicit instructions to direct the attention of observers to veridical kinematic information is questionable, particularly if disguised actions are performed. Future research could employ more implicit methods for attention cueing to ‘see through’ deceitful intent and explore the visual search strategies of observers viewing disguised action.

### Confidence Ratings

Perceptual judgements such as those performed when anticipating an opponent’s actions can be made with varying degrees of conscious awareness about how the decision was made [33, 40]. In some cases the observer can be very aware of how or why they are making the decision they do [40], while in other cases they may have less awareness, for instance if the response is embedded in movement [41] and/or if the dynamics of the action are directly specified by the action kinematics [8]. The measurement of an observer’s confidence when making perceptual judgements provides one possible way to make inferences about the degree of consciousness engaged when performing the task. *Higher Order Thought Theory* proposes that having thoughts about a state indicates that the state itself is conscious and can be inferred at different levels [42]. For example, an observer is understood to have greater confidence in their judgement if they have conscious awareness of the higher order thought level (cognitive processing) that may have taken place when performing the task [3]. Subsequently, high confidence ratings are thought to be associated with higher levels of cognitive processing whereas low confidence ratings are associated with judgments with less processing [43]. The use of confidence ratings can help to complement work on deception in sport by allowing inferences to be made about the degree to which deceptive actions elicit conscious processing on the part of the observer.

Smeeton and Williams [33] investigated the modes of processing associated with different confidence ratings. Skilled and less-skilled observers watched temporally occluded video footage of a football penalty taker performing deceitful, non-deceptive (veridical) and what they termed ‘non-deceptive-exaggerated kicks’ (i.e., those that went in the veridical direction but with exaggerated movements). Observers were required to anticipate the corner of the goal into which the ball was directed and to rate their confidence associated with that decision on a Likert scale from 1 (not at all confident) to 10 (extremely confident). Results revealed that skilled observers were more accurate than less-skilled observers at anticipating all kicking actions, with exaggerated kicks anticipated significantly better than non-deceptive kicks, which in turn were anticipated better than deceitful kicks across both groups. Confidence ratings indicated that skilled observers were significantly more confident than their less-skilled counterparts (also see [44] for loaded kicks). Across groups, confidence for the exaggerated kicks was rated higher than for the non-deceptive and deceitful kicks (also see [3] and [34] for contrasting results). Skilled observers were equally confident irrespective of the kicking action, but less-skilled observers recorded higher confidence ratings when viewing exaggerated and deceitful kicks compared to non-deceptive kicks. These results indicate that exaggeration in the kicking action engenders (sometimes false) confidence in less-skilled observers, with Smeeton and Williams [33] suggesting that this is associated with the detection of kinematic information that conveys false action intentions. From the perspective of Interpersonal Deception Theory, and in particular the role of intent when producing an action, this study shows it is possible for actors to exaggerate both veridical *and* deceitful action outcomes. And while Smeeton and Williams [33] referred to their exaggerated actions as ‘non-deceptive’, we argue that they actually should be classified as *deceptive* actions from the perspective of Interpersonal Deception Theory. The exaggerated actions were performed with the intent (i.e., the kickers were asked to deliberately alter their actions) to alter the perception of the observers viewing the actions. Therefore, they can be classified as deceptive, even though they convey the veridical action outcome. But does this make sense? Why should an exaggerated veridical action be classified as ‘deception?’ The most pertinent example of individuals performing exaggerated veridical actions for the sake of deception is for ‘dives’ in football and other team sports. In those situations, actors seek to convince a referee that the outcome of an opponent’s action was more severe than it really was in order to gain a foul or penalty call. Conceivably, exaggerated veridical actions could also help to deceive opponents rather than referees. For instance, an actor might want to engender overconfidence in an observer’s ability to pick-up on veridical movements to make their regular movements more disguised, or to enhance the deceptiveness of their exaggerated deceitful actions (i.e., to highlight the potential usefulness of kinematic information to the opponent). The usefulness of exaggerated veridical information in conveying deception appears a fruitful topic for future research.

Causer and Williams’s [14] study of different playing uniforms found that skilled observers recorded higher confidence levels than less-skilled players did, with higher confidence levels recorded for neutral (non-deceptive) shirts compared to a circled and zigzagged patterned (disguised) shirt. Together these studies suggest that a lower-level of cognitive processing is likely to be engaged when viewing disguised actions, and more so for non-deceptive actions [14, 33]. Whereas deceitful actions appear to invoke a higher-level of cognitive processing because exaggerated movements draw an observer’s visual attention, resulting in higher confidence ratings, uniforms designed to disguise actions, which are devoid of exaggerated movement, cause a drop in confidence.

### Contributions of Visual and Motor Experiences to the Perception of Deceptive Actions

The ability to anticipate the action outcome of an opponent can depend on both the visual *and* motor experience of the observer. Similarly, the ability of the actor to deceive the observer depends also on their visual and motor experience of the same action [26]. Prinz [45] in his *Common-coding Theory* proposed that a common representational domain underpins the ability to both perceive and perform that same action, suggesting that a plan to execute an action is activated when also viewing that action, and vice versa. Similarly, sensitivity for perceiving an action is facilitated when producing that same action. If true, the key implication of common-coding theory is that the ability of an actor to deceive an opponent could be improved by learning to better perform *or perceive* that movement, and vice versa, that the ability of an observer to better ‘see through’ deceptive intent could be improved by learning to perceive *or perform* that action.

Sebanz and Shiffrar [17] investigated the degree to which visual and motor expertise contribute to the perception of deceitful movements. Skilled and less-skilled observers were required to anticipate whether a basketball player would have passed a ball - or faked the pass - when viewing both dynamic and static images. Results revealed that the skilled players were significantly more accurate than the less-skilled players when viewing dynamic images, but not when viewing static images. The authors argued that skilled observers could detect deceitful movements because of their motor expertise in producing the same actions, though they could not entirely rule out the visual contribution to action perception because the skilled observers, through their playing experience, had no doubt viewed numerous passes and fake passes in the past.

Alternatively, Cañal-Bruland and Schmidt [32] examined the contribution of visual and motor experience to the perception of deceitful actions by testing two groups of skilled handball players (field players and goalkeepers) and a less-skilled group. They argued that field players had extensive motor *and* visual experience in performing and viewing both real and fake shots, whereas goalkeepers were considered to have had extensive visual but not motor experience performing the real and fake throws. Less-skilled observers had neither visual nor motor experience. Observers were required to detect whether a handball throw was real - or a baulk - when viewing video footage from a (neutral) side perspective. Results revealed that skilled observers (field players and goalkeepers) were more accurate at detecting deceitful actions than less-skilled observers, with no differences between the field players and goalkeepers. Given that both the field players and goalkeepers had unequal motor experience, the authors argued that the contribution of motor experience to action perception could not solely explain the expertise advantage in this experiment. Instead, the conclusion was that visual expertise best explained the ability of handball players to discriminate deceitful actions from genuine actions (also see [18]). Clearly, though, it remains possible that the goalkeepers had some degree of motor experience for performing the real and fake throws, and/or that skilled anticipation can be acquired through visual *or* motor experience [46].

More recently, Wright et al. [47] employed two sequential tasks to examine which brain regions were activated when viewing a deceitful action. While lying in an fMRI scanner, skilled and less-skilled observers watched point-light displays of skilled actors executing deceitful (step-over) and non-deceptive movements in Association Football. In the first task, observers were required to detect whether a deceitful action was present (deception identification task). Once completed, a second task required observers to anticipate the intended direction in which the actor would move (direction identification task). The results revealed that skilled observers showed significantly greater activation of the motor components of the action observation network than less-skilled observers (see [48, 49] for examples). Moreover, activation of the motor components of the action observation network was greater for the first (deception identification) task than for the second (direction identification) task. The greater activation of the action observation network when detecting deceitful action was said to be indicative of top-down cognitive and attentional control. Conversely, the reduced activation anticipating the intended direction highlights what could be more automatic perceptual task control. Interestingly the social network, which included the limbic region, was also activated more during the first task. This brain area is particularly activated when making inferences about other people’s intentions, suggesting that high levels of emotional intelligence could influence perceptual sensitivity to deception (see [50] for a relevant example).

Matching actors and observers has been challenging as interacting opponents differ in their degree of visual and motor experience of deceit and/or disguise, which may explain who will outperform who during the interplay of anticipation and deception. Future research designs could assess both the deceptiveness (i.e. deceit and/or disguise) when acting as a performer and the perceptual ability when observing the same deceptive action (e.g., see [28]). Alternatively, observers could view their own deceptive actions (e.g. self-other designs) where the deceptive action (i.e. deceit and/or disguise) being viewed resonates perfectly with the motor experience of the observer.

Finally, an understanding of Interpersonal Deception Theory and the mechanisms by which skilled actors perform deceptive actions can provide crucial guidance for training interventions designed to improve the ability to deceive others. First, it should be possible for an individual to improve the deceitfulness of their actions by training to perform or observe those same actions. Actors may need to learn to exaggerate their movements in the non-veridical direction to convey deceit, but perhaps not so much that it appears obvious and unrealistic to skilled observers [30]. Second, better disguised actions should be able to be learned by training to perform or observe those same actions. This may involve learning to deliberately wait as long as possible to perform a critical change of action or to minimize the degree to which the gaze direction specifies the action outcome. Lastly, the role of intent in deception suggests that it is possible through training to reduce the degree to which an individual’s regular veridical actions can be anticipated by opponents. The case of Pete Sampras’ training suggests that it is possible to use targeted training interventions to make even veridical action outcomes more difficult to anticipate. It would be interesting to discover whether the observation of those same difficult-to-anticipate actions would lead to the observer’s own actions becoming more difficult to anticipate.

## Factors Influencing Responses to Deceptive Actions

Evidence has shown that there is much more to real-world anticipatory performance than simply relying on information available from an opponent’s advanced kinematic cues. Skilled observers are also known to utilize *contextual* information as a complementary strategy for predicting the action intentions of interacting opponents [27, 51]. For example, skilled observers can identify and utilize their opponents’ action preferences such as their favoured action(s) in particular scenarios (e.g., for court position or game score) to assist in predicting action outcomes. This may also include knowledge about the likelihood of a deceptive action occurring [32]. In some senses this provides an extension of Interpersonal Deception Theory, given that, from that framework, the observer assumes that the interaction is likely to be honest. However, deceptive actions are instead common in competitive sports [36, 37], and so skilled observers may become more suspicious of their opponents’ intentions to act genuinely than less-skilled observers would. This suspicion may result in a higher expectation of deceptive intent, and this can lead to biases in the way that observers respond by having a higher expectation of the likelihood of deceptive intent based on their experience in the sport.

Cañal-Bruland and Schmidt [32] used *Signal Detection Theory* to investigate the response biases of skilled and less-skilled observers when discriminating between deceitful and non-deceptive handball throws. The skilled players (both goalkeepers and field players) had superior response accuracies and were more accurate at detecting deceitful actions than less-skilled observers (also see [26]), though evidently for different reasons. Signal Detection Theory analyses revealed that goalkeepers had a bias to judge shots as being fakes (i.e., deceitful) whereas field players did not have such a bias. The authors claimed that goalkeepers had adopted a more conservative approach to responding to shots-on-goal due to the acquisition of extensive knowledge of situational probabilities of deceitful and non-deceptive actions during penalty situations (fakes are very common in these scenarios) [52, 53]. This reflects the findings in other studies where skilled athletes are shown to have response biases that presumably reflect a functional adaptation to the likelihood of an event occurring (e.g., a baseball batter ‘sitting on a fastball’ [54]). Crucially, this provides an opportunity to extend on Interpersonal Deception Theory where there is typically an expectation of honesty in the interaction.

An observer’s action capabilities, such as their movement speed and agility, could also influence their strategy when responding to deceptive movements. Observers are afforded the opportunity to act in an environment [55], and the observer’s own capability to move will influence their response selection [25]. For example, Dicks et al. [56] investigated the ability of skilled goalkeepers to anticipate and successfully stop deceitful and non-deceptive shots-on-goal in an in-situ Association Football setting. Goalkeepers wore occlusion goggles that were opaque at the start of each trial. Visual information became available at various points in the penalty taker’s approach. The results revealed that goalkeepers generally initiated their responses early relative to ball-foot contact. However, during deceitful actions, goalkeepers exhibited significantly more response corrections to effect a save. The authors suggested that initiating movement responses earlier coincided with the presentation of deceitful actions which degraded goal-saving performance, unless the keeper could make a response correction that was quick enough to ultimately save the shot-on-goal. In a follow up study, Dicks et al. [25], using the same experimental set up, tested whether the movement speed of the goalkeeper was related to the success in stopping the shot. The results revealed that faster moving goalkeepers were more successful at stopping deceitful shots than were slow-moving goalkeepers. The authors suggested that slower moving goalkeepers had to move earlier to compensate for their slowness, which meant that they were likely to be responding to deceitful rather than veridical information [31]. In essence, the same ability to move late and fast that was generated for instance by Pete Sampras’ training could also benefit the ability to *respond* to deceptive actions. We will expand on the effects of action capabilities on perception in the section ‘Action Capabilities and Their Effect on Perception’.

Together, these findings suggest that skilled observers adapt their responses in a functional way according to the perceived likelihood of an outcome, the importance of the task, and the action capabilities of that observer. Skilled performance in deceptive situations requires performers to go beyond the assumption that deception will not occur, and to develop a realistic expectation that their opponents will perform a deceptive action. Suspicious behaviours of opponents may require skilled observers to utilise complementary contextual information to successfully predict action outcomes or alternatively, to allow observers to manipulate their acting opponent(s). Training the action capabilities of the observer may offer researchers an opportunity to overcome constraints on anticipatory performance in real-world tasks where the interplay between deception and anticipation is ongoing.

## Learning to Better Anticipate Deceptive Actions

Given that skilled observers exploit approaches that detect and anticipate deceptive actions, studies have attempted to implement training paradigms designed to improve the anticipation of deceptive actions. Crucially, the necessary training approach might differ depending on whether it is deceitful or disguised actions that are being observed. In one study examining deceit, Alsharji and Wade [57] used video-based perceptual training to improve the anticipatory skill of handball goalkeepers who face opponents who conveyed deceit through ‘baulked’ actions. Skilled penalty takers were filmed, from the goalkeepers’ perspective, executing both baulks (fakes) and non-deceptive throws. The footage was edited and temporally occluded one frame before ball-release. Goalkeepers watched the test videos on a life-sized screen and were required to anticipate the intended direction of the actions by moving as quickly as possible in that direction. Goalkeepers were then randomly assigned to one of three matched groups (train, placebo, and control) based on their anticipatory performance on the pre-test. For training, the train-group practiced by watching video clips of deceitful and non-deceptive actions on an electronic tablet. Three different video-clip conditions were produced for training: (i) temporal occlusion one frame before ball release while viewing the whole body of the penalty taker; (ii) temporal occlusion one frame before ball release while viewing only the upper body of the penalty taker and displayed at a speed of 25% slower than normal; and (iii) no occlusion, but a blinking arrow indicating the intended direction of the throw. The placebo-group practiced by watching real deceitful and non-deceptive actions from games without any editing of videos, while the control-group received no training. Training was performed for 20 min a day for seven consecutive days with no feedback provided during training. Results revealed that the train-group significantly improved their anticipatory performance while the placebo- and control-groups did not. Additionally, anticipatory performance was always significantly worse when anticipating deceitful rather than non-deceptive actions, even following training. Evidently it is possible to improve the ability to perceive deceit in opponents through exposure to video footage of those actions.

Attempts have been made to improve the ability to perceive deceitful intent through motor training, though the results have been so far mixed. For instance, Pizzera and Raab [21] investigated whether visual and/or motor training would improve the ability to detect ‘dives’ in Association Football. Note that dives can be classified as deceitful actions within the Interpersonal Deception Theory framework, but that they do constitute a special case given that they typically are exaggerated *veridical* actions. Skilled football players were randomly assigned into one of three groups (visual, motor, and control) and were required to make a forced-choice decision in a pre-post-retention video test. In the test, players watched the video, consisting of dives (deceitful actions) and fouls (non-deceptive actions) from television clips, and responded by pressing a corresponding button as quickly as possible. For training, the motor-group practiced their ability to perform one-on-one deceitful actions (i.e., exaggerated body checks, pretending to be kicked or tripped). Following each of eight practice sessions, the motor-group received feedback and were instructed on how to enhance the deceptiveness of their actions. The visual-group watched the motor-group from a referees’ viewing perspective, heard the feedback to enhance deceptiveness, and rated the deceptiveness of the motor-group’s actions. The control-group did not participate in any training intervention. Results revealed no change in the overall response accuracy of any of the three groups (also see [24, 58]). These results suggest that alternative methods should be employed to enhance the ability to ‘see through’ the intent to fake a foul in football (e.g., see [23] for a relevant example). Additionally, it is possible that the explicit instructions used to train the motor-group focussed too heavily on movement production rather than on the movement effect (see action-effect hypothesis [45]). Future studies could examine the effects of (non-visual or blindfolded) motor training (possibly using the action-effect hypothesis) and determine the perceptual sensitivity to deceitful and non-deceptive actions.

The application of Interpersonal Deception Theory highlights that attempts to train the ability to anticipate deceptive actions have focussed exclusively on deceitful actions, with no work yet as far as we know examining the training of *disguised* actions to better attune to contextual information that might help in the anticipation of deceptive intent (e.g., on the likelihood of deceptive actions [53]).

Moreover, studies so far have largely been conducted using video-based tests which eliminate or minimize the interaction between two opposing players [58]. There is a need for studies to examine whether training can genuinely enhance the ability to perceive and avoid deceptive intent in in-situ scenarios. Therefore, more representative in-situ designs that unlock the action capabilities of the observer and enhance the interactive nature between competing opponents should be considered in future research [1, 2]. Furthermore, assessing the transferability of enhanced deception perception into real-life game situations is imperative.

## Action Capabilities and Their Effect on Perception

**Fig. 2:**
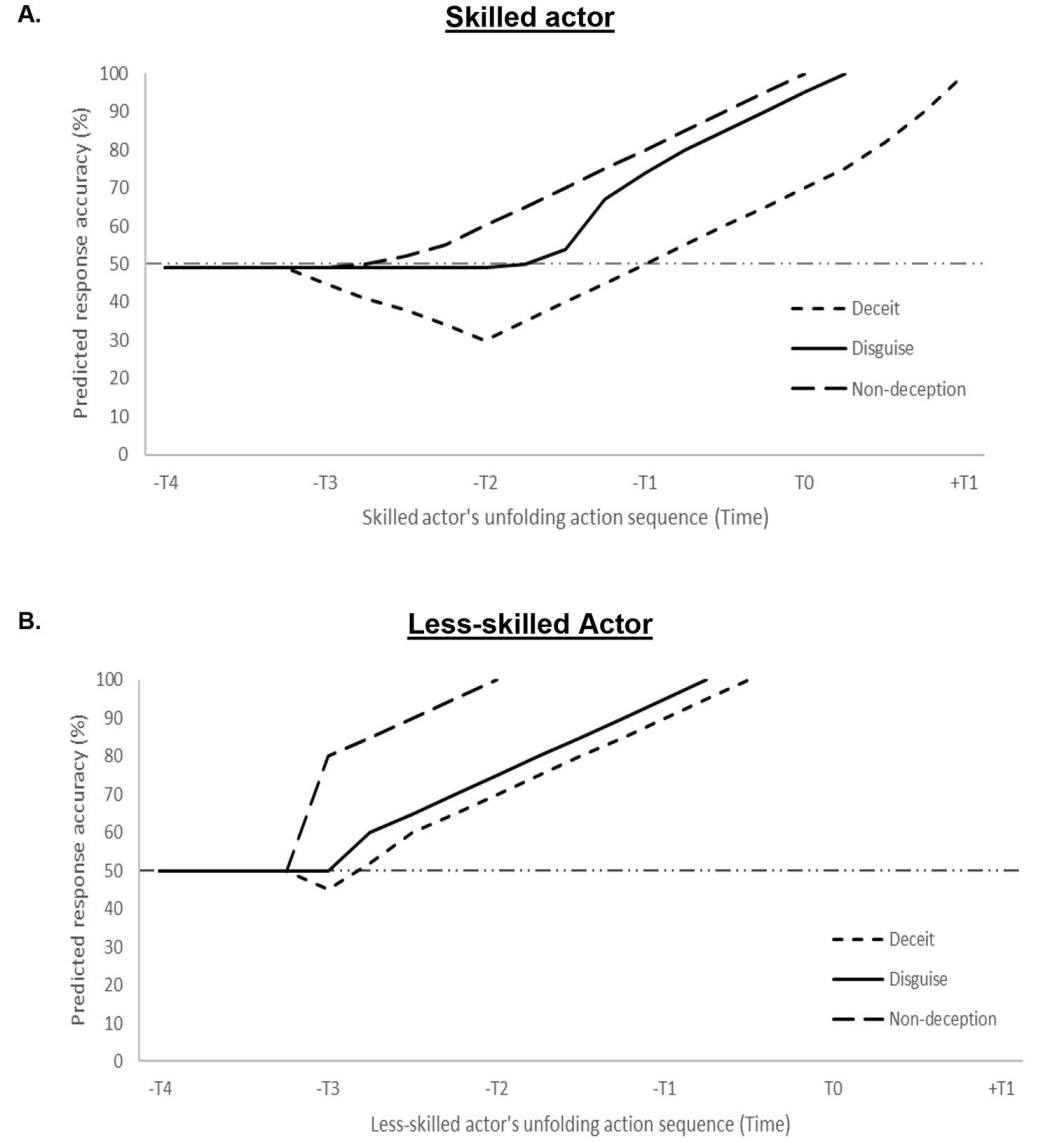
Predicted response accuracies when anticipating the deceitful, disguised, and non-deceptive actions of a (**A**) skilled actor and (**B**) less-skilled actor in an unfolding action sequence. Numbers on the y-axes represent hypothesised effects rather than actual calculations, with the numbers retained to show what are ceiling effects (0 & 100% response accuracy) and chance guessing (50%) in a two-alternative forced-choice paradigm

Skilled and less-skilled performers differ in their ability to perform authentically deceptive actions. In particular, it may be more difficult to perceive the deceptive actions of a skilled performer than it is for a less-skilled performer, especially when that action is occluded earlier in an unfolding action sequence [3]. In this section, we bring together the insights from Interpersonal Deception Theory to put forward hypotheses and graphically illustrate what the response accuracy of an observer might look like when attempting to anticipate the (deceptive) action intentions of skilled and less-skilled performers (see Fig. 2). As a result, we put forward a series of empirically testable predictions.

*Non-deceptive (veridical) actions*. When viewing non-deceptive actions, veridical information about the action intention becomes increasingly obvious as the action sequence unfolds. We expect that this veridical information will become available later in the action sequence when observing the actions of skilled when compared to less-skilled actors [3, 12] (see Fig. 2A vs. B). We postulate that this skill-based difference is best explained by the differences in the action capabilities of skilled actors that allow them to initiate the action later in the action sequence than less-skilled actors. Accordingly, response accuracy when predicting the action of a skilled actor would be equal to or worse than that when predicting the action of a less skilled actor irrespective of the moment in the action sequence. In a sense, the skilled actor’s action could be understood as being ‘well-hidden’ or even ‘disguised’, though when viewed from the Interpersonal Deception Theory, the action is not classified as a disguised action given that there is no intent on the part of the skilled actor to disguise their action. Training that improves an actor’s action capabilities should, in principle, lead to impairments in the ability of an observer to predict the action outcome.

*Disguised actions*. When observing disguised actions, veridical information about the action intention should become available *later* in the action sequence (see solid lines in Fig. 2A & B). Having suggested that skilled actors may present veridical information later even in non-deceptive trials, we expect that skilled actors, when deliberately disguising their actions, will be further able to delay their opponent’s ability to anticipate their true action intention.

*Deceitful actions*. When observing deceitful actions, veridical movement information will become available *later* in the action sequence following the presentation of non-veridical movement information (see short-dashed lines in Fig. 2A & B). We expect that skilled actors would be better able to present exaggerated movements that genuinely simulate an outcome that is in conflict with that which will ultimately be the actual outcome or with what was the cause of the action (e.g., in the case of a dive in football). Moreover, they will do so at time points in the action sequence that correspond with what would be expected if actually producing that ultimately incorrect action outcome. These types of movements are only possible when the action capabilities of the skilled actor allow them to re-orientate their movements late in the action without compromising the desired action outcome. Deceitful actions generally drop prediction accuracy below chance levels (see [5, 31, 56]). Given the action capabilities of skilled actors, prediction accuracies are expected to remain below chance-level until later in the action sequence than what would be possible when observing less-skilled actors. The observer’s own action capabilities will dictate whether they still have enough time to respond effectively.

## Conclusion

The aim of this paper was to outline a conceptual framework for understanding deceptive actions in sport. The conceptual framework defined deception as an action that impairs the perception of the observer and adopts Interpersonal Deception Theory to make an unambiguous distinction between disguise and deceit. Moreover, deceptive actions are those that are performed with intent, and therefore it is vital when deciding whether an action is deceptive to consider whether the actor intended for the action to be deceptive, irrespective of whether or not the observer was actually deceived. Empirical evidence shows that skilled observers are less susceptible to deceptive actions than less-skilled observers, but that deception does reduce the anticipatory performance of all observers. Skilled observers have acquired and developed specialised underlying mechanisms to become more sensitive to deceptive actions, but training studies designed to improve the ability to anticipate the outcome of deceptive actions have so far demonstrated limited success and focussed exclusively on deceit rather than disguise. Finally, hypotheses were put forward to predict how the anticipatory performances of observers would be affected by different types of deception, and by actors with different skill levels. Future research should focus on deception in in-situ settings, helping to unlock the action capabilities of the observer and to establish the transferability of perceptual training to improve the on-field perception of deception.

## Data Availability

Not applicable.
